# Bladder cavernous hemangioma after pelvic radiotherapy in a female patient: A case report and literature review

**DOI:** 10.1016/j.ijscr.2018.11.044

**Published:** 2018-11-24

**Authors:** Xinming Hu, Kangli Deng

**Affiliations:** a Department of Urology, The Second Affiliated Hospital of Hainan Medical University, Haikou, 570100, PR China; b Department of Urology, Hubei Cancer Hospital, Tongji Medical College, Huazhong University of Science and Technology, Wuhan, 430079, PR China

**Keywords:** Bladder carvenous hemangioma, Case report, Hematuria, Radiotherapy, Transurethral resection

## Abstract

**Introduction:**

Hemangiomas are benign tumor formations of capillaries and blood vessels which are commonly found in various organs. However they are extremely rare in urinary bladder accounting for only 0.6% of all urinary bladder tumors. The differentiating of these benign tumors from malignant neoplasms are important since they have extremely different prognostic features as well as therapeutic strategies. Here we reported a female patient diagnosed with bladder cavernous hemangioma (BCH) after recent pelvic radiotherapy for the cervical cancer and reviewed relevant literatures.

**Case presentation:**

We reported a case of 49 years old female patient with persistent painless hematuria for 12 days. Computed tomography revealed a small lesion on the superior wall of the urinary bladder with acute clot retention. Cystoscopy confirmed a solid papillary pedunculated mass with a measuring of 1.0 × 0.5 cm located on the superior posterior wall and surrounded by distended vessels. Transurethral resection of the mass was then performed and the pathological report indicated a cavernous hemangioma of the urinary bladder. In a one and half year follow-up, no tumor recurrence or bleeding was found.

**Conclusions:**

The cavernous hemangioma in urinary bladder is rare and cystoscopic is a gold standard for diagnosis. Treatment options are vary for individuals and pathologic findings are vital for differentiating it from malignant potential tumors. A history of cancer related radiation therapy seems to be a risk factor for BCH.

## Highlights

• The bladder cavernous hemangioma (BCH) is a benign non-urothelial tumor rarely occurred in the urinary bladder.
• Treatment options are vary for individuals and most are with favorable follow-ups.
• A history of cancer related radiation therapy seems to be a risk factor for BCH.
• It is important to differentiate them from malignant neoplasms since they have extremely different prognostic features and therapeutic strategies.

## 1 Introduction

Hemangiomas are benign vascular tumors which are common mesenchymal tumors in soft tissue and can be found in various organs [1]. However, hemangioma of the urinary bladder is very rare and only accounts for 0.6% of all bladder tumors [1,2]. Hemangiomas are usually small in the bladder and nearly 80% were cavernous type [2]. The most common presenting symptom is mild hematuria, with or without suprapubic pain due to vesical irritation and urinary retention [3]. Transurethral endoscopic surgery is the gold standard for the treatment of bladder cavernous hemangioma (BCH) although vary for individuals, and follow-ups show favorable outcomes in several studies [1,[3], [4], [5]].

It is important to carefully differentiate benign hematuria from malignant neoplasms such as angiosarcoma and Kaposi sarcoma, which are associated with immunosuppressed patients, since they have vital different prognostic features [2]. Nevertheless, several studies have confirmed increased risks of developing soft tissue tumor in relation to radiation therapy for cancer [6]. In this article we present a female patient diagnosed with BCH after recent pelvic radiotherapy for the cervical carcinoma and histological features consistent with the diagnosis of BCH after transurethral resection of the tumor. This case has been reported in linewith the SCARE criteria [7].

## 2 Case presentation

**Fig. 1:**
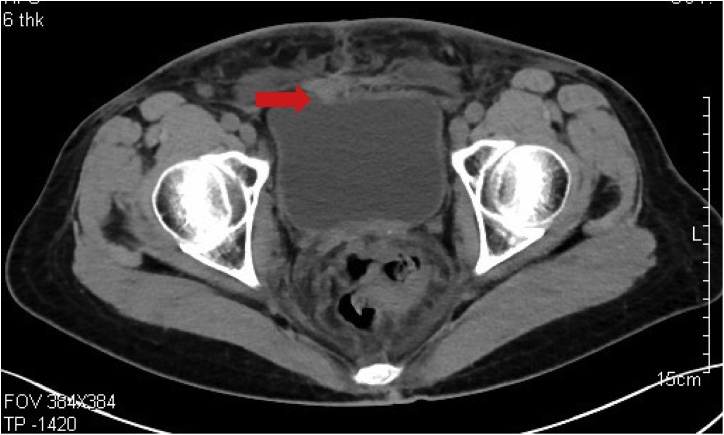
Computed tomography revealed a small lesion on the superior wall of the urinary bladder.

**Fig. 2:**
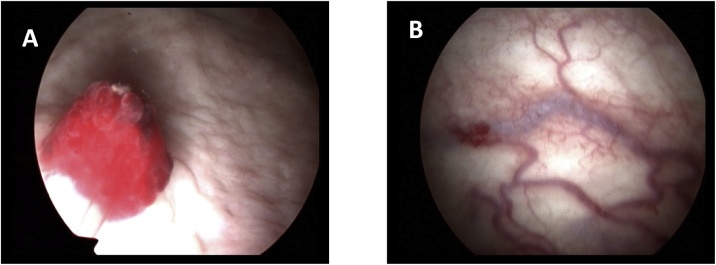
(A): Cystoscopy revealed a solid papillary pedunculated mass with a measuring of 1.0 × 0.5 cm located on the superior posterior wall. (B): The surrounding urinary wall have several distended vessels.

A 49 years old female was referred to a sudden, painless hematuria 12 days prior for her visit to our hospital. She had past history of cervical cancer about one year ago and received surgery as well as adjuvant chemotherapy and radiotherapy thereafter. The latest follow-up showed no evidence of tumor recurrence. Physical examination revealed no palpable mass or enlarged lymphnodes. She had also diagnosed with diabetes type 2 for 10 years with no medication pills treatment. Computed tomography revealed a small lesion on the superior wall of the urinary bladder with acute clot retention (Fig. 1). Cystoscopy confirmed a solid papillary pedunculated mass with a measuring of 1.0 x 0.5 cm located on the superior posterior wall (Fig. 2A). The surface of the mass revealed reddish and vascular formation. The surrounding urinary wall have several distended vessels which seems to be associated with the hemangioma formation (Fig. 2B).

**Fig. 3:**
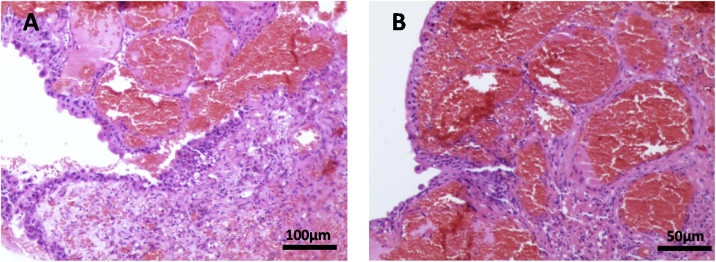
(A) Histological findings revealed the almina propria and submucosa of the urinary wall without infiltration of the muscularis propria. (B) A proliferation of vessel walls with distinct borders and spreading between the normal vasculature, well differentiated, and the stroma of the bladder submucosa with intense congestion.

We then performed transurethral tumor resection and the tissue sample was sent to the pathological examination. Histological findings revealed the almina propria and submucosa of the urinary wall without infiltration of the muscularis propria (Fig. 3A). They were found to be as a proliferation of vessel walls with distinct borders and spreading between the normal vasculature, well differentiated, and the stroma of the bladder submucosa with intense congestion (Fig. 3B). The pathological diagnosis was bladder hemangioma cavernous type according to the histological features described above. The symptom of hematuria disappeared after the surgery and no evidence of tumor recurrence was found in one year and half follow-ups.

## 3 Discussion

Most bladder tumors arise from the urothelium, and non-urothelial neoplasms are very rare in the bladder [1]. These include both benign and malignant lesions [8]. Benign non-urothelial neoplasms including leiomyoma, hemangioma, neurofibroma, and schwannoma, and in particular, hemangioma is the most common lesion among the series [2]. For the malignant non-urethelial bladder tumors, including squamous cell carcinoma, adenocarcinoma and sarcoma, usually referred to poor prognostic and needs to be carefully differentiated [9].

Hemangiomas are benign tumors formatted by capillaries and blood vessels, and the most common hemangiomas occurred in bladder is cavernous type, while much less frequent are capillary or arteriovenous types [2]. Up to now there is only a few reported cases worldwide on hemangiomas of the urinary bladder [1,4,5,[10], [11], [12], [13]]. Bladder hemangiomas is typically small, ranged from 0.5 cm to 3 cm, or >5 cm although even rare, affecting the dome, posterior wall, or trigone of the bladder [1]. Although it can occur in any age of individuals, the most often is under 30 years of age and slightly more common among men [3]. The histologic depth of a bladder hemangioma may be within the submucosa, however extension to the muscular layer or even to the perivesical tissues is not uncommon [1,2]. Multiple bladder hemangiomas may be associated with the Klipper-Trenaunay-Weber and Sturge-Weber syndromes, predisposing to their development [2,3]. The most common symptom is gross hematuria with or without irritative urinary symptoms and abdominal pain [3].

Ultrasonography is the first-line imaging method for the evaluation of gross hematuria, while other imaging tests such as computed tomography and magnetic resonance imaging are also useful in the diagnosis of vascular mass [5]. The cystoscopic features varies from small punctuate areas to cherry-like raised lesions to large polypoid formations [2]. The main differential diagnoses for pigmented lesions seen on gross under endoscopy include endometriosis, melanoma, and sarcoma [14].

Since it is not commonly seen BCH in genitourinary tract, pathologists and doctors need to carefully differentiate it from malignant non-urothelial neoplasms, as they have extremely different prognostic features as well as therapeutic strategies [2,14]. The major differential diagnosis of BCH is malignant vascular tumor, such as angiosarcoma, which is highly aggressive potential with the characterizations of infiltrative growth, clear cytological atypia, high cellularity and poor prognosis [9]. On the contrary, BCH is typically characterized by proliferated of vessel walls with distinct borders and spreading between the normal vasculature, and which lack distinct endothelial atypia or multilayering and with favorable prognosis [2]. In this case, the patient received radiotherapy recently for cervical cancer, as radiation therapy is well known predisposing factor to the development of angiosarcomas, hence a history of radiation seems to be a risk factor for BCH, as reported previously [6,[15], [16], [17]].

The therapeutic approaches for the management of BCH are vary in individuals due to their size, location and depth of penetration. Optional strategies includes observation, transurethral resection, electrocoagulation, radiation, systemic steroid administration, sclerosing agent injection, and partial cystectomy [3,12]. Partial cystectomy and radiation therapy are effective for >3 cm masses or multiple tumors [1]. For small tumors, like in this case, transurethral resection has become the standard surgical intervention [3]. The majority of the clinical follow-up of BCH reported previously are favorable, and in this case no evidence of recurrence occurred during the one and half year follow-ups [1,3].

## 4 Conclusions

The BCH is a benign non-urothelial tumor rarely occurred in the urinary bladder and treatment options are vary for individuals with favorable follow-ups. A history of cancer related radiation therapy seems to be a risk factor for BCH.

## Conflicts of interest

None.

## Sources of funding

This work was supported by grants from National Natural Science Foundation of China (grant number 81700669), the Natural Science Foundation of Hubei Province of China (grant number 2016CFB217), and the China Scholarship Council.

## Ethical approval

This study was approved by Ethics Committee of Hubei Cancer Hospital.

## Consent

Written informed consent was obtained from the patient for publication of this case report and accompanying images. A copy of the written consent is available for review by the Editor-in-Chief of this journal on request.

## Author contribution

Xinming Hu – data collection, data analysis or interpretation, writing the paper.

Kangli Deng – study concept or design, data collection, data analysis or interpretation, writing the paper.

## Registration of research studies

As this was a case report and not a clinical trial, this study does not require registration.

## Guarantor

Kangli Deng will be the corresponding author and take the full responsibility for the work and/or the conduct of the study, had access to the data and controlled the decision to publish.

## Provenance and peer review

Not commissioned, externally peer reviewed.
